# Pharmaceutical Services for Visually Impaired Patients: Views and Challenges Perceived by Pharmacists and Patients in the Qassim Region of Saudi Arabia

**DOI:** 10.3390/healthcare14050567

**Published:** 2026-02-25

**Authors:** Layan Ali Alkhoshaiban, Layan Khalid Alolayan, Shahad Salah Aleid, Reham Almutiri, Norah Aljulaydan, Abddulmajeed Shoieb Alharbi, Mohammed Saif Anaam, Waleed M. Altowayan, Abdulrahman A. Alsuhaibani, Saud Alsahali

**Affiliations:** 1 Department of Pharmacy Practice, College of Pharmacy, Qassim University, Buraydah 51452, Qassim, Saudi Arabia411202190@qu.edu.sa (S.S.A.); aa.alsuhibani@qu.edu.sa (A.A.A.); 2 Department of Pharmaceutical Care, King Fahd Specialist Hospital in Buraydah, Qassim Health Cluster, Buraydah 52366, Qassim, Saudi Arabia

**Keywords:** pharmaceutical services, knowledge, visually impaired patients, survey, community pharmacy, Saudi Arabia

## Abstract

**Background:** Visual impairment (VI) describes decreased visual function that interferes with an individual’s ability to perform daily activities, such as reading, driving, and other executive tasks. Providing optimal pharmaceutical care for this population can be challenging for pharmacists, as individuals with VI face numerous obstacles in managing their medications. This study explores the challenges experienced by pharmacists and visually impaired patients and aims to compare their points of view to identify existing gaps and propose recommendations to optimize medication use among individuals with VI. **Method:** A cross-sectional study was conducted between September 2024 and January 2025 among pharmacists (n = 152) and visually impaired patients (n = 31) in the Qassim Region of Saudi Arabia. Pharmacists completed a self-administered questionnaire, while data from visually impaired patients were collected via structured face-to-face interviews. Data analysis was performed using IBM SPSS Version 21, with a significance level set at *p* < 0.05. **Results:** A total of 152 pharmacists and 31 visually impaired individuals participated in the study. Only 5.3% of pharmacists had received training related to VI care, and most counseling was directed to caregivers rather than patients (80.3%). pharmacists with additional training in VI were significantly more likely to employ unique or appropriate packaging methods (*p* < 0.001), and male pharmacists were more likely to rely on caregivers during OTC counseling (*p* = 0.014). Among the visually impaired participants, 77.5% reported difficulty reading medication packages and 67.7% faced challenges entering pharmacies. Braille literacy was significantly higher among males (*p* = 0.018) and those with higher education (*p* = 0.022). Overall, 90.3% expressed a need for improved accessibility tools and communication support in pharmacies. **Conclusions:** Pharmacists showed confidence in assisting visually impaired patients; however, most lacked formal training and relied heavily on caregivers for communication. Visually impaired individuals also reported difficulties accessing pharmacies, reading medication labels, and receiving complete information from pharmacists. Based on these findings, implementing specialized training programs, expanding Braille and tactile labeling, and integrating assistive technologies within pharmacies are recommended to improve safety and equitable medication use.

## 1. Pharmaceutical Services for Visually Impaired Patients: Views and Challenges Perceived by Pharmacists and Patients in the Qassim Region of Saudi Arabia

Visual impairment (VI) describes decreased visual function that interferes with an individual’s ability to perform daily activities, such as reading, driving, and other executive tasks [1]. Individuals with total blindness are those who have a complete lack of light perception (no light perception; NLP). About 15% of individuals with eye diseases have total blindness, while the majority of those with VI have some level of vision [1]. Globally, VI represents a growing public health concern with profound social and healthcare implications. In Saudi Arabia, the General Authority for Statistics’ disability survey states that more than 40,000 people have seeing difficulties, with 1057 people in the Qassim Region having extreme vision difficulties [2]. This prevalence reinforces the need to guarantee safe and effective medication for all individuals with VI [3]. Providing optimal counseling and managing medications for these patients can be challenging for both pharmacists and visually impaired patients. Pharmacists have reported several challenges, including difficulty recognizing patients with VI, dependence on caregivers or family members, communication barriers with visually impaired patients, and various limitations, such as limited time, high workloads, and restricted access to patient records, which hinder their ability to provide optimal pharmaceutical care [4,5,6,7]. Meanwhile, visually impaired individuals face numerous challenges in their medication management, including identifying and organizing medicines, the accidental loss or splitting of tablets, a limited ability to detect dispensing errors, and challenges in adhering to prescribed treatment courses [8]. These barriers can compromise medication safety and therapeutic outcomes, underscoring the critical role of pharmacists in supporting this vulnerable population.

A 2018 study conducted in South Korea assessed medication use and pharmacy services for visually impaired individuals by incorporating perspectives from both community pharmacists and visually impaired persons. The findings revealed that most visually impaired individuals encountered difficulties in identifying medications at home but adapted through personal strategies, such as using distinct storage containers or designated locations for differentiation. Furthermore, participants expressed a preference for more detailed counseling and the use of assistive tools during medication education. However, most pharmacists reported providing counseling primarily to caregivers or family members rather than directly to visually impaired patients [7]. A study conducted in 2020 investigated medication use patterns among visually impaired individuals in Saudi Arabia, emphasizing the need for Braille labeling and stated that the current method of dispensing for this population were not sufficient to meet the need of health information [9]. More recently, in 2024, another study explored the challenges and supportive factors in medication dispensing and counseling for visually impaired patients, based on pharmacists’ perspectives and the results highlighted the need of improve medication counselling for patients with vision impairment [4].

Despite these efforts, there remains a lack of comprehensive research examining both medication use and pharmacy counseling for visually impaired individuals in Saudi Arabia, particularly studies that integrate the viewpoints of both pharmacists and visually impaired patients. Addressing this gap is essential to improve pharmacy services and ensure equitable access to safe and effective medication for use for this population. Therefore, this study aimed to explore the views and challenges that pharmacists face in their current daily practice. In addition, the study aimed to explore medication use and counseling practices among visually impaired individuals in Saudi Arabia. By comparing the perspectives of pharmacists and visually impaired patients, this research seeks to identify the existing gaps and propose evidence-based recommendations on how pharmacists can better support safe and optimized medication use for individuals with VI.

## 2. Methodology

### 2.1. Study Population and Sampling Method

This study was designed as a prospective, cross-sectional study conducted among pharmacists and visually impaired individuals in the Qassim Region of Saudi Arabia. A convenience sampling approach was utilized for participant recruitment. For the pharmacist cohort, data collectors visited a series of selected pharmacies, including hospital-based pharmacies. Each data collector provided a brief explanation of the study’s aims and procedures before sending the questionnaire to the participant. Participation was voluntary, and informed consent was obtained from each pharmacist prior to completion. The data privacy and confidentiality for the participants were secured. Recruitment was limited to a fixed four-month period (September 2024 to January 2025), which resulted in a final sample of 152 pharmacists.

Data from individuals with VIs were collected through face-to-face interviews, facilitated with the assistance of a local charity association, Mubseron which provide many help services for VI people. With the support of Mubseron, data collectors attended the Saudi 95th national day celebration event organized by the association, which was attended by a diverse group varying in gender, age, and place of residence. During this event, the data collectors conducted the interviews. The data collection procedure involved the researchers reading the questions and corresponding response options aloud to the participants, who then indicated their choices. The researchers subsequently recorded the selected answers. At the beginning of each interview, the researcher explained the study’s objectives and obtained the participant’s verbal informed consent. The modest sample size of visually impaired participants (n = 31) reflects the practical constraints of recruiting this specific, low-prevalence population within a single region, despite proactive recruitment through a key community organization.

### 2.2. Data Collection Tool

The questionnaire used in this study was adapted from a Korean study, in which a questionnaire was developed using appropriate methods and validated for reliability [7]. Permission for reuse was obtained from the original authors, and the original English questionnaire was translated into Arabic by a bilingual researcher and then independently back-translated by a second bilingual researcher to verify semantic equivalence. The final Arabic version was reviewed for clarity and cultural appropriateness by the research team prior to data collection.

For the pharmacists, the survey questionnaire consisted of four sections and a total of 18 questions: baseline demographics (four questions), the use of pharmacy services by visually impaired patients (four questions), medication counseling for visually impaired patients (seven questions), and adverse drug events reported by visually impaired patients (two questions). At the end of the survey, an additional open-ended question was included to capture pharmacists’ suggestions for improving pharmacy services to enhance convenience and accessibility for patients with VI.

For individuals with VI, the questionnaire comprised three main categories: baseline demographics (nine questions), the use of medications (nine questions), and pharmacy services utilization (five items), for a total of 23 items.

### 2.3. Data Entry and Analysis

The data were entered into Microsoft Excel for Microsoft 365 (Microsoft, Redmond, WA, USA) and analyzed using IBM SPSS Statistics, Version 21 (IBM Corp., Armonk, NY, USA). Categorical data are presented as frequencies and percentages. The relationships between variables were assessed using the chi-squared test, with a *p*-value of less than 0.05 considered statistically significant. This analysis was an exploratory, descriptive analysis aimed at identifying patterns and associations between variables, rather than testing pre-specified hypotheses.

## 3. Results

### 3.1. Pharmacists

Out of 152 participants, male pharmacists constituted 86.8% of participants, while females represented 13.2% (Table 1). Most pharmacists had more than 10 years of experience (30.9%), followed by those with 4–6 years (23.7%) and 7–10 years (21.1%). The majority were employed in community pharmacies (73.7%), compared to 19.7% in government hospitals and 6.6% in private hospitals. Regionally, Buraydah (34.2%) and Unaizah (30.9%) accounted for the highest representation among respondents across the Qassim Region.

As shown in Table 2, 90.1% of pharmacists reported receiving 1–5 visually impaired patients monthly, while only 3.9% received more than 10. Braille requests were infrequent, with 88.8% reporting “rarely.” Only 5.3% of respondents had received specific training on visually impaired care. In terms of confidence, 77.6% of pharmacists reported being “fairly confident” or “very confident” when counseling visually impaired patients.

The most common dispensing practice was using the original packaging (84.2%), followed by unique packaging (11.2%) (Table 3). For prescription medications, 80.3% of pharmacists reported counseling family members or caregivers, whereas only 10.5% used specialized tools to aid visually impaired patients. In OTC counseling without Braille labeling, 84.9% relied on family or caregivers, indicating that most communication with visually impaired patients occurs indirectly through companions rather than directly.

Only 9.9% of pharmacists reported encountering an adverse drug reaction (ADR) among visually impaired patients, and just 3.3% reported any medication-related incident (Table 4).

Two statistically significant associations were identified (Table 5). First, pharmacists with additional training in VI were significantly more likely to employ unique or appropriate packaging methods (*p* < 0.001). Second, a significant association was found between gender and the OTC counseling approach, with male pharmacists more frequently relying on caregivers than their female counterparts (*p* = 0.014). All other analyzed relationships were non-significant (*p* > 0.05).

As shown in Table 6, pharmacists’ responses to open-ended questions revealed notable trends in their professional experiences and perspectives regarding visually impaired patients. The majority (39.5%) identified visually impaired individuals primarily through caregiver accompaniment, while 30.9% recognized them through self-reporting.

Regarding the most common nonprescription medications, vitamins and supplements (42.8%) and pain relievers (30.9%) were the most frequently dispensed items, reflecting the reliance on easily accessible OTC products by visually impaired patients.

In terms of pharmacists’ learning needs, understanding patient needs (75.7%), communication and counseling skills (60.5%), and Braille labeling knowledge (59.9%) were prioritized.

Finally, the most frequently reported adverse drug events were linked to chronic disease medications (51.6%).

**Figure 1:**
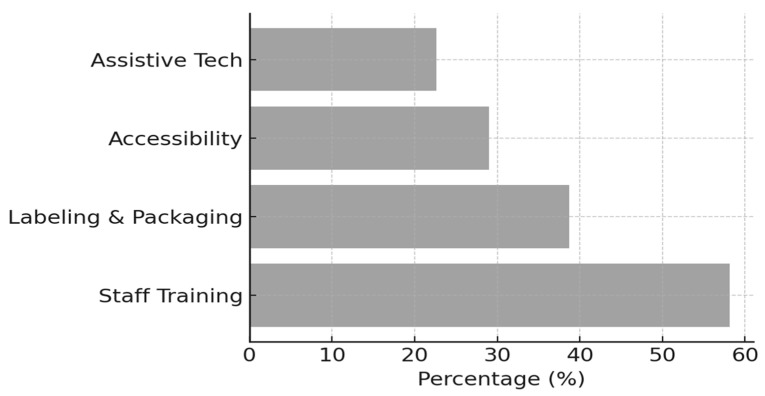
Pharmacists’ Suggestions to Improve Accessibility for Visually Impaired Patients.

As shown in Figure 1, the most frequently suggested improvement was the need for specialized staff training (58.1%). The second most common suggestion was enhancing labeling and packaging (38.7%), particularly through the use of Braille labels, tactile identifiers, and clear print to reduce medication errors. Additionally, 29.0% of pharmacists emphasized the importance of improving pharmacy accessibility, such as clearer navigation and physical space adjustments, while 22.6% highlighted the integration of assistive technologies, including mobile apps and voice-based instructions, to support medication adherence and safety.

### 3.2. Visually Impaired Participants

Male participants represented 54.8% of the visually impaired group, and most were aged between 20 and 40 years (74.2%) (Table 7). The majority resided in Buraydah (74.2%) and lived with family members (93.5%). A large proportion (61.3%) received government assistance. Regarding their visual condition, 51.6% were totally blind and unable to distinguish light. More than half (51.6%) held a bachelor’s degree, and notably, the same proportion (51.6%) were able to read Braille.

Two-thirds of the visually impaired participants (67.7%) reported difficulty entering pharmacies (Table 8). The most common method to facilitate medication use was receiving help from family members (41.9%), while 32.3% reported not using any specific method. Reading medication packages was challenging for most, with 77.5% experiencing some level of difficulty. Regarding confidence, 48.4% felt confident handling medications independently, whereas only 6.5% were not confident. The majority (54.8%) disposed of leftover medications properly, although 45.2% kept them for later use. The most frequently cited OTC challenges were identifying dosages and timings (32.3%) and understanding side effects and expiry dates (25.8%).

As shown in Table 9, 64.5% of participants reported receiving verbal counseling from pharmacists, and 67.7% stated that the pharmacist provided clear instructions. However, only 22.6% mentioned being informed about possible side effects. Most participants had a clear (61.3%) or partial (29.0%) understanding of their medications after counseling. In terms of satisfaction, 45.2% felt neutral. A large majority (90.3%) expressed a need for more accessibility tools in pharmacies, highlighting a strong unmet demand for assistive communication strategies. Verbal explanation was the preferred method of counseling for visually impaired patients (58.1%).

Most participants (87.1%) had not experienced any medication errors, while 12.9% reported at least one incident (Table 10). However, only 6.5% reported these events to a healthcare professional, suggesting underreporting. Nearly one in five (19.4%) admitted to taking the wrong medication at least once, and 35.5% had missed a dose unintentionally. The most common preventive strategy was caregiver or family reminders (58.1%), followed by organizing medications using schedules or pillboxes (19.4%). Overall, 58.1% expressed confidence in their medication safety behaviors.

Two statistically significant relationships were observed. The analysis of gender and the ability to read Braille (*p* = 0.018) indicated that male participants were more likely to be Braille literate, while analysis of education level and the ability to read Braille (*p* = 0.022) indicated that patients with higher education levels had greater Braille proficiency.

## 4. Discussion

This study explored the current pharmaceutical services provided for visually impaired people in the Qassim Region of Saudi Arabia and the practices among pharmacists serving this population. Furthermore, the study evaluated the needs and challenges faced by patients with VI. Integrating these perspectives addresses a gap in the existing evidence in Saudi Arabia.

### 4.1. Pharmacists

The study found that VI in patients was primarily identified by pharmacists through the accompaniment of a caregiver (39.5%) or the patient’s self-reporting (30.9%) rather than through any systematic assessment. These results match those observed in the previous literature, where nondisclosure by visually impaired patients is common (Merenda et al., 2024) [10], whereas Kentab et al. (2024) [4] showed that pharmacists in Saudi Arabia reported hesitation toward direct inquiry due to sensitivity concerns. In sum, relying on speculative clues or voluntary self-reporting contributes to the under-recognition of visually impaired patients and limits the consistent provision of tailored pharmaceutical care. Encouraging pharmacists to use proactive, structured approaches to inquire about disability, while fostering a supportive environment for patient disclosure, may help close this recognition gap and improve accessibility-focused care.

Consistent with Lee and Lee (2019) [7], who found that 61% of counseling was directed toward family members or caregivers, our study found that in both prescription and over-the-counter medications, pharmacists relied on family members and caregivers for counseling, reported at 80.3% and 84.9%, respectively. This implies that medication counseling is mainly provided through a mediator rather than directly, which limits opportunities for pharmacists to engage with the patients themselves. However, this may introduce various communication risks, including information being incomplete, filtered, modified, or inaccurately relayed by the caregiver, or the pharmacist not receiving the patient’s feedback or questions. Evidence from South Africa revealed that by communicating with visually impaired patients, pharmacists had a better understanding of their needs: significant medication information was given, and they were able to devise ways to make medical products accessible [11]. It is also important to mention that the minimal use of specialized counseling tools in our sample (10.5%) might further reinforce a cycle where the family or caregiver are the default communication channel.

Braille is a tactile system that utilizes raised dots to represent letters and numbers, enabling individuals with VI to read and write independently [12,13,14]. In total, 88.8% of pharmacists reported that requests for Braille-labeled medications were rare. Of particular interest is that although some patients in our sample were literate in Braille, the Braille text was mostly printed in English, creating a language barrier that further constrained the practical use of Braille in medication management. The interplay of low patient exposure and Braille-related language barriers may collectively limit patients’ independent access to medication information, thereby increasing their reliance on caregivers and potentially affecting adherence and autonomy. In response to this issue, the Saudi Food and Drug Authority released a guideline in 2023 mandating that Braille be printed in Arabic on outer medication packaging; however, this has not yet been implemented [15,16].

The data from this study indicate a fundamental gap in specialized training related to visual impairment care, with only 5.3% of pharmacists reporting formal instruction for this population. Previous literature demonstrated similar findings of limited or absent tailored training [4,10]. In contrast, most pharmacists (77.6%) stated confidence in counseling visually impaired patients, which likely stems from general clinical expertise rather than a particular proficiency in providing pharmaceutical care for this patient population. The observed association between formal training and the deployment of modified packaging and incorporated communication strategies (*p* > 0.001) suggests that even limited, targeted education might be associated with better practices, although causality cannot be inferred. All things considered, depending solely on experience is exiguous in meeting visually impaired patients’ particular accessibility and safety needs, emphasizing the need to embed structured training through both initial and ongoing professional education.

### 4.2. Visually Impaired Patients

The evidence from this study showed that pharmacies had substantial and unacceptable shortfalls in physical accessibility, with 67% of participants reporting difficulties entering the pharmacy. This finding is congruent with data from Ethiopia and the World Health Organization demonstrating consistent structural barriers within pharmacy environments [17,18]. It is probable that this physical inaccessibility partially explains the modest encounter rates by this population, as 90.1% of pharmacists in our sample revealed encountering only one to five visually impaired patients per month, a pattern similarly seen in South Korea [7]. To resolve this deficit, directed environmental refinements are needed, including step-free entrances, sufficient and uniformly distributed lighting meeting higher illumination requirements for people with sight loss [19,20], and clear and effective wayfinding systems. Other points worth mentioning are the implementation of tactile signage within reachable height ranges, using embossed rather than engraved characters [20], as well as the incorporation of Braille on all packages and price labels. These modifications may promote safe, independent navigation and advance equity in pharmaceutical care.

Our study demonstrates a constant reliance on intersubjective support and the scarce use of supportive tools among adults with VI managing their medications. In particular, 41.9% of patients reported receiving help from caregivers, whereas 32.3% did not use any particular method to organize or track their medications. These findings are consistent with other research across many countries, which found that reliance on caregivers continues to be a prevalent approach in this population [9,21,22], and the greater part of participants in a study conducted in Malaysia (65%) denied utilization of any commercially available assistive devices [23]. This may reflect underlying challenges in accessibility, awareness, or the usability of assistive technologies, suggesting that current support may not fully enable independent medication management.

In this study, the participants frequently reported difficulties in identifying information on medications, particularly regarding dosages and timings (32.3%) and understanding side effects or expiry dates (25.8%), with 77.5% experiencing challenges in reading packaging. Consistent with previous research, many individuals with VI require caregiver support for medication recognition, with 26% to 96.5% reporting identification difficulties in various settings [9,21,23,24]. Incorporating touch cues on packaging, using dosette boxes to organize pills by time of day, and adhering to large-print, sans-serif labeling guidelines (minimum 12–14 pt, avoiding all capitals or underlining) can guide correct use and enhance independence [25,26,27].

The data revealed particular patterns in how visually impaired patients navigate and coordinate routine medication use. Almost one-fifth (19.4%) stated that they had taken the wrong medication, while above one-third (35.5%) inadvertently missed doses, demonstrating that daily tasks can be disrupted despite patients’ familiarity with their regimens. Management practices appeared to rely predominantly on interpersonal support, as caregiver or family reminders were the main strategy (58.1%), whereas more structured self-management tools, such as schedules or pillboxes, were used by only 19.4% of patients with VI. This pattern suggests that external assistance compensates for, but may not fully resolve, the underlying accessibility challenges in medication handling for this population.

### 4.3. Strengths and Limitations of the Study

This study’s key strengths are its novel dual-perspective design in Saudi Arabia, its use of a pre-validated data collection tool, and its successful recruitment of a hard-to-reach patient population through a community partnership, which yielded rich, mixed-methods data on a critically underserved group.

However, the study is also subject to several limitations. The use of a convenience sampling method for both pharmacists and visually impaired patients, coupled with the small sample size of the patient cohort (n = 31), may affect the generalizability of the findings. Furthermore, the significant gender imbalance in the pharmacist sample (86.8% of respondents were male) limits the interpretation and extension of gender-based results. Finally, the self-reported nature of the surveys introduces the potential for social desirability bias; pharmacists may have over-reported their confidence and adherence to best practices, while patients may have under-reported dissatisfaction or medication-related errors.

Notwithstanding these limitations, the concurrent examination of both patient and pharmacist perspectives offers valuable, foundational insights into critical gaps in training, communication, and accessibility that impact medication safety and independence. These findings can inform health policy, pharmacy education, and the development of more inclusive pharmacy practices. To build upon these findings, future research should prioritize international comparative studies to identify and evaluate best practices in pharmaceutical care for visually impaired patients. Specifically, there is a need for interventional studies that assess the impact of structured pharmacist training programs on patient outcomes, as well as research evaluating the effectiveness and implementation of assistive technologies and devices in reducing medication-use errors within this population. In addition, future studies should employ random sampling strategies with larger, more diverse populations to enhance generalizability.

## 5. Conclusions

This study aimed to explore and compare the perspectives of pharmacists and visually impaired patients in the Qassim Region of Saudi Arabia regarding medication use and counseling practices. The findings revealed that although most pharmacists expressed confidence in assisting visually impaired patients, only a small proportion had received formal training, and counseling was predominantly directed toward caregivers rather than the patients themselves. Conversely, most visually impaired individuals reported difficulties in reading medication labels, accessing pharmacies, and receiving complete information about their medications. This disparity underscores a significant gap between the perceived adequacy of pharmacy services and the actual needs of visually impaired patients, emphasizing the importance of accessibility, patient-centered communication, and equitable pharmaceutical care. To address these gaps, it is recommended that pharmacists receive specialized training in counseling visually impaired patients, that the nationwide implementation of Braille and tactile medication labeling be adopted, and that the integration of assistive digital technologies, such as voice-based applications, is promoted to enhance medication safety, adherence, and patient independence. Also, the pharmaceutical industry could play pivotal role in helping people with VI through making the products packaging more friendly for people with VI.

## Figures and Tables

**Table 1:** Demographic and Professional Characteristics of Pharmacists (n = 152).

Variable	Category	Frequency (n)	Percentage (%)
Gender	Male	132	86.8
	Female	20	13.2
Years of Experience	<1 year	5	3.3
	1–3 years	32	21.1
	4–6 years	36	23.7
	7–10 years	32	21.1
	>10 years	47	30.9
Work Sector	Government Hospital	30	19.7
	Private Hospital	10	6.6
	Community Pharmacy	112	73.7
Region (Qassim)	Buraydah	52	34.2
	Unaizah	47	30.9
	Al-Baday’	13	8.6
	Al-Bukayriyah	11	7.2
	Al-Rass	17	11.2
	Al-Mudhnab	6	3.9
	Al-Nabhaniyah	3	2.0
	Uyun Al-Jawa	3	2.0

**Table 2:** Exposure to Visually Impaired (VI) Patients, Training, and Confidence Level Among Pharmacists (n = 152).

Variable	Category	Frequency (n)	Percentage (%)
Monthly VI Patient Visits	None	1	0.7
	1–5 patients	137	90.1
	6–10 patients	8	5.3
	>10 patients	6	3.9
Frequency of Braille Requests	Every visit	5	3.3
	Often	12	7.9
	Rarely	135	88.8
Additional Training on VI Care	Yes	8	5.3
	No	144	94.7
Confidence in Counseling VI Patients	Not confident at all	1	0.7
	Not very confident	4	2.6
	Neutral	29	19.1
	Fairly confident	45	29.6
	Very confident	73	48.0

**Table 3:** Dispensing and Counseling Practices Among Pharmacists (n = 152).

Variable	Category	Frequency (n)	Percentage (%)
Dispensing Practices	Use original packaging	128	84.2
	Use unique packaging	17	11.2
	Printed plastic bags	1	0.7
	Electronic prescription	1	0.7
	Conversation with companion	1	0.7
	Use appropriate packaging	2	1.3
	None	2	1.3
Prescription Counseling (Rx)	To family/caregivers	122	80.3
	Use discrimination tools	16	10.5
	Frequency of consultation	8	5.3
	No VI clients	2	1.3
	Other	4	2.6
OTC Counseling (No Braille)	Consult family/caregivers	129	84.9
	Repeat consultation	13	8.6
	Use special containers	4	2.6
	Never	1	0.7
	Other	5	3.3

**Table 4:** Safety Outcomes Among Pharmacists (n = 152).

Variable	Category	Frequency (n)	Percentage (%)
Reported Adverse Drug Reaction (ADR)	Yes	15	9.9
	No	137	90.1
Reported Medication-Related Incident	Yes	5	3.3
	No	147	96.7

**Table 5:** Crosstab Analysis between Key Variables among Pharmacists (n = 152).

Crosstab Comparison	Statistical Test	*p*-Value	Notes/Interpretation
Region × Monthly VI patient visits	Pearson χ^2^ (df = 21)	0.985	Not significant
Sector × Monthly VI patient visits	Pearson χ^2^ (df = 6)	0.422	Not significant
Advice method × Years of experience	Pearson χ^2^ (df = 16)	0.592	Not significant
ADR (ever) × Years of experience	Pearson χ^2^ (df = 4)	0.464	Not significant
Dispense method × VI-related training	Pearson χ^2^ (df = 6)	0.000	Significant
Monthly VI visits × Gender	Pearson χ^2^ (df = 3)	0.685	Not significant
Dispense method × Gender	Pearson χ^2^ (df = 6)	0.110	Not significant
Training × Gender	Pearson χ^2^ (df = 1)	0.258	Not significant
Confidence × Gender	Pearson χ^2^ (df = 4)	0.275	Not significant
ADR (ever) × Gender	Pearson χ^2^ (df = 1)	0.433	Not significant
OTC counseling (no Braille) × Gender	Pearson χ^2^ (df = 4)	0.014	Significant

**Table 6:** Open-Ended Questions and Response Options Among Pharmacists (n = 152).

Question	Response Options	Frequency (n)	Percentage (%)
How do you know if a patient has visual impairment?	When the patient is accompanied by a caregiver	60	39.5
	The patient self-reports visual impairment	47	30.9
	By observing behavior/verbal cues	26	17.1
	From medical records or prescriptions	19	12.5
What non-prescription medications are most often used by visually impaired patients?	Vitamins and supplements	65	42.8
	Pain relievers	47	30.9
	Flu and cold medicines	24	15.8
	Eye drops	16	10.5
What knowledge do you need to acquire as a pharmacist to better serve visually impaired patients?	Understanding patients’ needs	115	75.7
	Braille labeling and packaging knowledge	91	59.9
	Communication and counseling techniques	92	60.5
	Legal/regulatory awareness	73	48.0
If yes, what type of medication(s) was reported in adverse drug events?	Chronic disease medications (e.g., diabetes, hypertension)	16	51.6
	Pain relievers	8	25.8
	Supplements	5	16.1
	Allergy medications	2	6.5

**Table 7:** Demographic and Clinical Characteristics of Visually Impaired Patients (n = 31).

Variable	Category	Frequency (n)	Percentage (%)
Gender	Male	17	54.8
	Female	14	45.2
Age (years)	20–30	12	38.7
	31–40	11	35.5
	41–50	7	22.6
	51–60	1	3.2
Residence (Qassim)	Buraydah	23	74.2
	Unaizah	2	6.5
	Al-Baday’	1	3.2
	Al-Bukayriyah	1	3.2
	Al-Rass	1	3.2
	Other	3	9.7
Living Status	Alone	2	6.5
	With Family/Roommate	29	93.5
Receiving Government Assistance	Yes	19	61.3
	No	12	38.7
Description of Visual Condition	Total blindness—cannot distinguish light	16	51.6
	Blindness—can only distinguish light	11	35.5
	Severe VI—can see shapes/objects	3	9.7
	Blindness—can distinguish movement	1	3.2
Educational Level	Intermediate or lower	5	16.1
	Secondary school	9	29.0
	Bachelor’s degree	16	51.6
	Master’s or PhD	1	3.2
Ability to Read Braille	Yes	16	51.6
	No	15	48.4

**Table 8:** Pharmacy Access, Medication Use, and Challenges Among Visually Impaired Patients (n = 31).

Variable	Category	Frequency (n)	Percentage (%)
Difficulty Entering Pharmacy	Yes	21	67.7
	No	10	32.3
Ways to Help Use Medications	Getting help from family	13	41.9
	Use different containers or locations	3	9.7
	Braille packaging	3	9.7
	Touch and package	2	6.5
	No specific methods	10	32.3
Ease of Reading Medication Package	No difficulty	7	22.6
	Somewhat difficult	14	45.2
	Find difficulty	10	32.3
Handling Leftover Medications	Keep and use later	14	45.2
	Dispose of them	17	54.8
Confidence in Handling Medications Independently	Not confident	2	6.5
	Somewhat confident	14	45.2
	Confident	15	48.4
Most Difficult Challenges with OTC Medications	Medication recognition problems	5	16.1
	Difficulty identifying timings/dosages	10	32.3
	Difficulty identifying side effects/expiry dates	8	25.8
	Multiple difficulties combined	8	25.8

**Table 9:** Communication and Counseling Experience with Pharmacists Among Visually Impaired Patients (n = 31).

Variable	Category	Frequency (n)	Percentage (%)
Receiving Verbal Counseling by Pharmacist	Yes	20	64.5
	No	11	35.5
Satisfaction with Verbal Counseling	Satisfied	11	35.5
	Neutral	14	45.2
	Dissatisfied	6	19.4
Pharmacist Provided Clear Instructions	Yes	21	67.7
	No	10	32.3
Understanding of Medication Purpose After Counseling	Clear understanding	19	61.3
	Partial understanding	9	29.0
	Did not understand	3	9.7
Pharmacist Mentioned Possible Side Effects	Yes	7	22.6
	No	24	77.4
Preferred Method of Counseling	Verbal explanation	18	58.1
	Written label or Braille	4	12.9
	Assistance from family member	9	29.0
Need for More Accessibility Tools in Pharmacies	Yes	28	90.3
	No	3	9.7

**Table 10:** Safety Behavior and Medication Adherence Among Visually Impaired Patients (n = 31).

Variable	Category	Frequency (n)	Percentage (%)
Ever Experienced a Medication Error	Yes	4	12.9
	No	27	87.1
Reported the Error to Pharmacist or Doctor	Yes	2	6.5
	No	29	93.5
Ever Took Wrong Medication by Mistake	Yes	6	19.4
	No	25	80.6
Ever Missed a Dose Accidentally	Yes	11	35.5
	No	20	64.5
Methods Used to Prevent Medication Errors	Family or caregiver reminders	18	58.1
	Using tactile markers (e.g., rubber bands)	5	16.1
	Organized schedule or pillbox	6	19.4
	Mobile phone alarms	2	6.5
Confidence in Medication Safety Practices	Not confident	3	9.7
	Somewhat confident	10	32.3
	Confident	18	58.1

## Data Availability

The datasets used for this study were available from the corresponding author on reasonable request.
